# Systemic inequalities in indoor air pollution exposure in London, UK

**DOI:** 10.5334/bc.100

**Published:** 2021-05-07

**Authors:** Lauren Ferguson, Jonathon Taylor, Ke Zhou, Clive Shrubsole, Phil Symonds, Mike Davies, Sani Dimitroulopoulou

**Affiliations:** UCL Institute for Environmental Design and Engineering, The Bartlett School of Environment Energy and Resources, University College London, London, UK; UCL Institute for Environmental Design and Engineering, The Bartlett School of Environment Energy and Resources, University College London, London, UK; Department of Civil Engineering, Tampere University, Tampere, Finland; UCL Institute for Environmental Design and Engineering, The Bartlett School of Environment Energy and Resources, University College London, London, UK; Environmental Hazards and Emergencies Department, Public Health England, UK. UCL Institute for Environmental Design and Engineering, The Bartlett School of Environment Energy and Resources, University College London, London, UK; UCL Institute for Environmental Design and Engineering, The Bartlett School of Environment Energy and Resources, University College London, London, UK; UCL Institute for Environmental Design and Engineering, The Bartlett School of Environment Energy and Resources, University College London, London, UK; Environmental Hazards and Emergencies Department, Public Health England, UK

**Keywords:** air quality, deprivation, environmental health, housing, indoor air pollution, inequalities, particulates, public health, systems thinking

## Abstract

Deprived communities in many cities are exposed to higher levels of outdoor air pollution, and there is increasing evidence of similar disparities for indoor air pollution exposure. There is a need to understand the drivers for this exposure disparity in order to develop effective interventions aimed at improving population health and reducing health inequities. With a focus on London, UK, this paper assembles evidence to examine why indoor exposure to PM_2.5_, NO_x_ and CO may disproportionately impact low-income groups. In particular, five factors are explored, namely: housing location and ambient outdoor levels of pollution; housing characteristics, including ventilation properties and internal sources of pollution; occupant behaviours; time spent indoors; and underlying health conditions. Evidence is drawn from various sources, including building physics models, modelled outdoor air pollution levels, time–activity surveys, housing stock surveys, geographical data, and peer-reviewed research. A systems framework is then proposed to integrate these factors, highlighting how exposure to high levels of indoor air pollution in low-income homes is in large part due to factors beyond the control of occupants, and is therefore an area of systemic inequality.

**Policy Relevance:**

There is increasing public and political awareness of the impact of air pollution on public health. Strong scientific evidence links exposure to air pollution with morbidity and mortality. Deprived communities may be more affected, however, with limited evidence on how deprivation may influence their personal exposure to air pollution, both outdoors and indoors. This paper describes different factors that may lead to low-income households being exposed to higher levels of indoor air pollution than the general population, using available data and models for London (*i.e*. living in areas of higher outdoor air pollution, in poor-quality housing, undertaking more pollution-generating activities indoors and spending more time indoors). A systems approach is used to show how these factors lead to systemic exposure inequalities, with low-income households having limited opportunities to improve their indoor air quality. This paper can inform actions and public policies to reduce environmental health inequalities, considering both indoor and outdoor air.

## 1 Introduction

Air pollution exposure is the greatest environmental health threat in the UK, with long-term exposures estimated to cause 28,000–36,000 premature deaths a year (COMEAP 2018). It is associated with several negative health outcomes, for example, respiratory (Atkinson *et al*. 2016) and cardiovascular complications (Arden Pope *et al*. 2011), birth defects (Padula *et al*. 2013), childhood asthma cases and sudden infant deaths (NICE 2013). Less explored is the association between air pollution exposure and mental illnesses, where long-term exposure to particulate matter (PM) has been associated with adult depression (Braithwaite *et al*. 2019) and children growing up in areas with high concentrations of nitrogen dioxide (NO_2_) may have an increased likelihood of developing schizophrenia (Horsdal *et al*. 2019). A critical review of the epidemiological evidence suggests that air pollution adversely affects cognitive function and is associated with cognitive impairment and increased risk of dementia (Delgado-Saborit *et al*. 2020). Upwards of 9000 people living in London die prematurely due to elevated levels of PM_2.5_ and NO_2_ each year, and the health impacts from exposure are estimated to cost the London economy between £1.4 billion and £3.7 billion annually (Walton *et al*. 2015).

In environmental health, the term ‘triple jeopardy’ can refer to the idea that low-socioeconomic status (SES) communities are (1) exposed to greater environmental hazards, such as air pollution, (2) have an increased susceptibility to poor health outcomes due to pre-existing health burdens such as chronic stress, poorer health status and less opportunity to choose health-promoting behaviours, which (3) result in health disparities across SES groups (Hajat *et al*. 2015). A recent systematic review investigated the relationship between exposure to outdoor air pollution and social inequalities in Europe (Fairburn *et al*. 2019). Some studies have found a positive association between outdoor air pollution and deprivation (Samoli *et al*. 2019), while others have suggested this association depends on the city-specific infrastructure (Temam *et al*. 2017).

In England, the health inequalities gap increased between 2001 and 2016 (Bennett *et al*. 2018) and the risk of death from preventable health conditions is three times higher for those living in the poorest areas of the country compared with individuals in the least deprived areas (ONS 2019c). Though preventing and responding to Covid-19 outbreaks is the current priority for Public Health England (PHE) (DHSC 2020), the 2019 PHE remit letter on reducing health inequalities via preventative measures cited exposure to air pollution as one of the leading causes of preventable health problems (DHSC 2019).

Most research into air pollution exposure inequalities relies on outdoor air pollution levels and census-level population indicators. However, in the UK—as with all other developed countries—the population spends around 90% of their time indoors (Lader *et al*. 2006), making buildings, and homes in particular, important microenvironments for air pollution exposure (Smith *et al*. 2016). There is growing evidence of indoor exposure disparities for particulate pollution and nitrogen oxides (NO_x_) between income groups in developed countries (Ferguson *et al*. 2020). Domestic indoor air pollution can be impacted by various factors, such as indoor sources, occupant behaviour, housing ventilation and local ambient levels of air pollution (RCPCH 2020; Shrubsole *et al*. 2016; Taylor *et al*. 2014, 2019), and changes to these that can modify indoor air pollution exposures. Ten years on from the landmark Marmot Review into health inequalities (Marmot 2010), Marmot advocates for the creation of healthy and sustainable places, via improvements in the quality of the built environment and air pollution concentrations, as one means of addressing the increase in health inequalities seen over the last decade (Marmot 2020).

The present paper discusses five important reasons why those in low SES groups may on average be exposed to greater levels of indoor air pollution in the domestic environment, focusing on London and the pollutants PM_2.5_, NO_x_ and CO. To illustrate how these factors may lead to exposure disparities, this paper:

- models indoor air pollution data using building physics tools
- performs Geographic Information System (GIS) analyses on outdoor air pollution data, proximity to busy roads and local deprivation indices
- analyses time-use and building stock data surveys and
- reviews other relevant studies.

These factors are then linked together using a systems analysis to show how systemic inequalities reinforce unequal exposures, with limited opportunities for low-income households to directly improve their own indoor air quality (IAQ).

## 2 Factors Driving Indoor Air Pollution Exposure Disparities

### 2.1 Outdoor Air Pollution Levels Are Often Higher In Deprived Areas

Research from North America (Clark *et al*. 2017; Pinault *et al*. 2016), Europe (Fairburn *et al*. 2019; Samoli *et al*. 2019) and parts of Southeast Asia (Choi *et al*. 2016; Li *et al*. 2018) have shown that low socioeconomic areas can have elevated concentrations of outdoor air pollution. However, environmental inequalities can show high levels of inter-city variation, and the relationship between area deprivation and outdoor concentrations depends on city-specific infrastructure and socioeconomic make-up. Research from highly urbanised areas such as Paris (Padilla *et al*. 2014) and New York (Hajat *et al*. 2013) indicates that areas of higher SES may have higher levels of outdoor air pollution due to wealthier individuals seeking the convenience offered by closer proximity to urban services. Since urban cores are characterised by high concentrations of traffic-generated air pollution (Padilla *et al*. 2014; Tonne *et al*. 2018), this can result in affluent areas subject to the highest levels of air pollution.

In some areas of the UK, outdoor air pollution often displays a ‘U’-shaped exposure pattern where both the *most* and the *least* deprived communities are exposed to elevated outdoor concentrations of pollutants (Brunt *et al*. 2017). Areas of low SES in the UK predominantly experience the highest levels of air pollution, but the strength and direction of this association can vary from one city to another. Outdoor concentration disparities between SES groups have been identified across areas of England (Barnes *et al*. 2019; Brainard *et al*. 2002; Fecht *et al*. 2015; Milojevic *et al*. 2017) and Wales (Barnes *et al*. 2019; Brunt *et al*. 2017).

While there have been considerable recent improvements to ambient air quality (GLA 2020b), London experiences high levels of air pollution, with levels of PM and NO_2_ often exceeding European Union (EU) limits (London Councils 2018). Table 1 displays a sample of relevant studies examining SES inequalities to outdoor air pollution in London. Inequalities are particularly pronounced in London compared with other areas of the country, where NO_2_ concentrations were 7.8 μg/m^3^ higher in the most deprived neighbourhoods compared with the least deprived neighbourhoods (Fecht *et al*. 2015). Annual average NO_x_ levels in London were found to be elevated in areas of the city with higher neighbourhood deprivation scores (Goodman *et al*. 2011) and outdoor residential NO_2_ and PM_2.5_ concentrations were 1.3 and 0.12 μg/m^3^ lower, respectively, for homes with the highest household income compared with the lowest (Tonne *et al*. 2018). Differences between study results may be attributable to the use of different metrics for SES and models using concentration or exposure.

**Figure 1:**
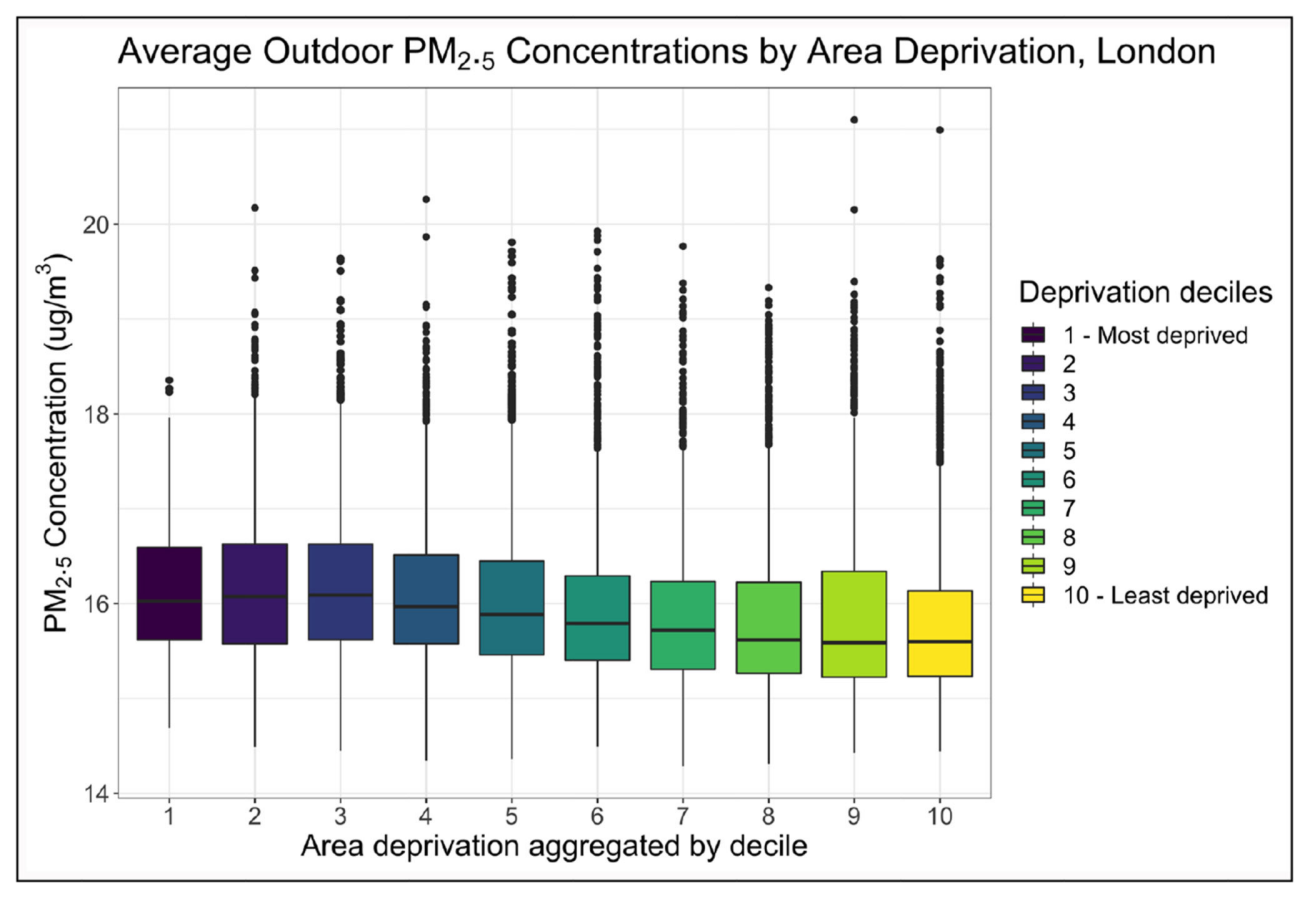
Annual average modelled outdoor PM_2.5_ concentration aggregated by area-deprivation decile for London Lower Super Output Areas (LSOAs).

Figure 1 illustrates how average outdoor PM_2.5_ concentration varies with deprivation in London. Using GIS, modelled annual average gridded (1 × 1 km) outdoor PM_2.5_ estimates (GLA 2017) were spatially overlaid with Lower Super Output Area (LSOA) boundaries. LSOA-average PM_2.5_ levels were then linked to the corresponding LSOA Indices of Multiple Deprivation (IMD) data (ONS 2019b), and the distributions of concentrations shown.^1^ Results support previous research, indicating higher exposures in areas of lower SES relative to areas with higher SES. Median PM_2.5_ concentration for areas in the lowest deprivation decile were 15.6 compared with 16.0 μg/m^3^ for those in the most deprived decile. Whilst studies consistently find more deprived areas are subject to worse outdoor concentrations of PM, the relatively small difference shown here affirms the limited spatial variation of PM_2.5_ across London due to its abundance of sources (Tonne *et al*. 2018), in contrast to NO_2_ which is primarily emitted by diesel vehicles.

Various studies have suggested that road traffic-related air pollution disproportionately impacts the most deprived areas (Brook & King 2017; Padilla *et al*. 2014; Tonne *et al*. 2018). Traffic is an important source of air pollution in London. For example, 50% of all NO_2_ emitted in the capital in 2015 was from diesel vehicles (London Councils 2018). Proximity to traffic may cause land or house prices to depreciate, attracting purchase or rental by low-income individuals and local councils for social housing (Deguen & Zmirou-Navier 2010).

**Figure 2:**
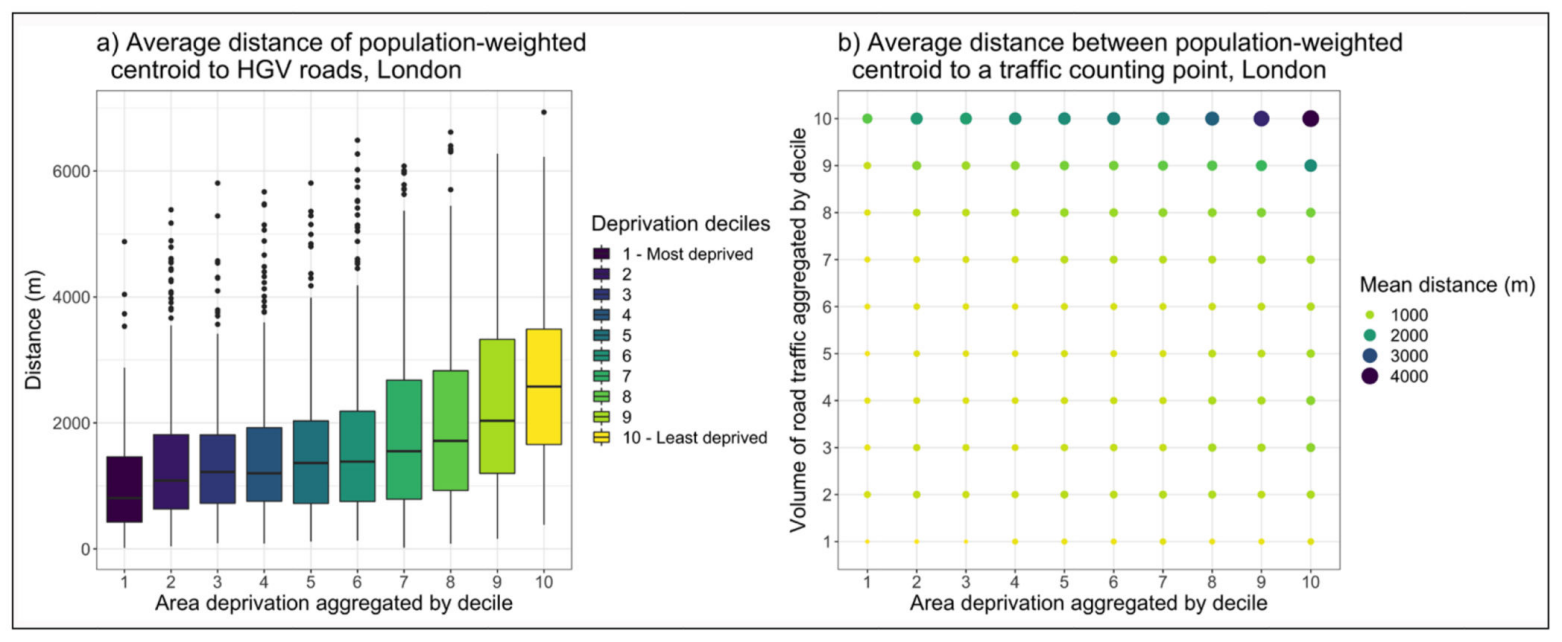
Average distance between Lower Super Output Area (LSOA) population-weighted centroid, aggregated by LSOA deprivation decile **(a)** traffic counting points in the top quartile for heavy goods vehicle (HGV) traffic; and **(b)** traffic counting points aggregated by deciles for all road traffic.

To illustrate how proximity to busy roads may differ according to SES, GIS was used to analyse the distance of London LSOAs to road traffic counting points data from the Department for Transport (DfT) (2020). Figure 2(a) shows a novel analysis of the distance from each LSOA population-weighted centroid to road traffic count points in the top quartile for heavy goods vehicle (HGV) traffic, by LSOA IMD. Such roads are typically characterised by higher traffic densities and greater levels of atmospheric pollution (Font & Fuller 2016). It shows that LSOAs with the highest levels of area deprivation are, on average, closer in proximity to roads with the greatest amounts of HGV traffic. Figure 2(b) shows the average proximity of LSOA population-weighted centroids to different deciles of total road traffic, by IMD decile. It shows a clear increase in average distance between LSOAs and busy roads as the IMD decile increases. These analyses are also reflected in the assessments of the areas surrounding dwellings in London in the English Housing Survey (EHS) (DCLG 2011), which indicates that the lowest income quintile households are more likely to live by a major trunk road or main road (19%) than the highest quintile (14%).

The varying proximity to busy transport routes raises questions around environmental justice. In general, low-income households are also the least likely to be contributing to outdoor air pollution in London, or benefitting from the production of the pollution. For example, Barnes *et al*. (2019) found areas of England and Wales with the highest proportion of households living in poverty were exposed to elevated concentrations of traffic-related air pollution relative to more affluent areas, despite low-income areas generally having lower levels of car ownership.

### 2.2 Inadequate Housing Can Increase Indoor Air Pollution Exposures

Houses in the UK are typically mixed mode, relying on both natural ventilation and infiltration for background ventilation, and extractor fans to remove indoor-generated pollutants and moisture when cooking or showering. The ventilation strategy for new buildings is specified in Approved Document F (HM Government 2013) and uses an air permeability threshold where naturally ventilated dwellings that do not meet this threshold will require a fixed amount of purpose-provided ventilation. The ventilation of a building can modify air pollution exposures both by the rate at which outdoor air pollution can infiltrate via cracks, flues, chimneys and vents in the building’s structure, and by the rate of removal of indoor-generated pollution through ventilation. The extent of dwelling infiltration is determined by the permeability and area of the exposed facades, exposure of the building to the wind and the presence of background ventilation features such as chimneys.

**Figure 3:**
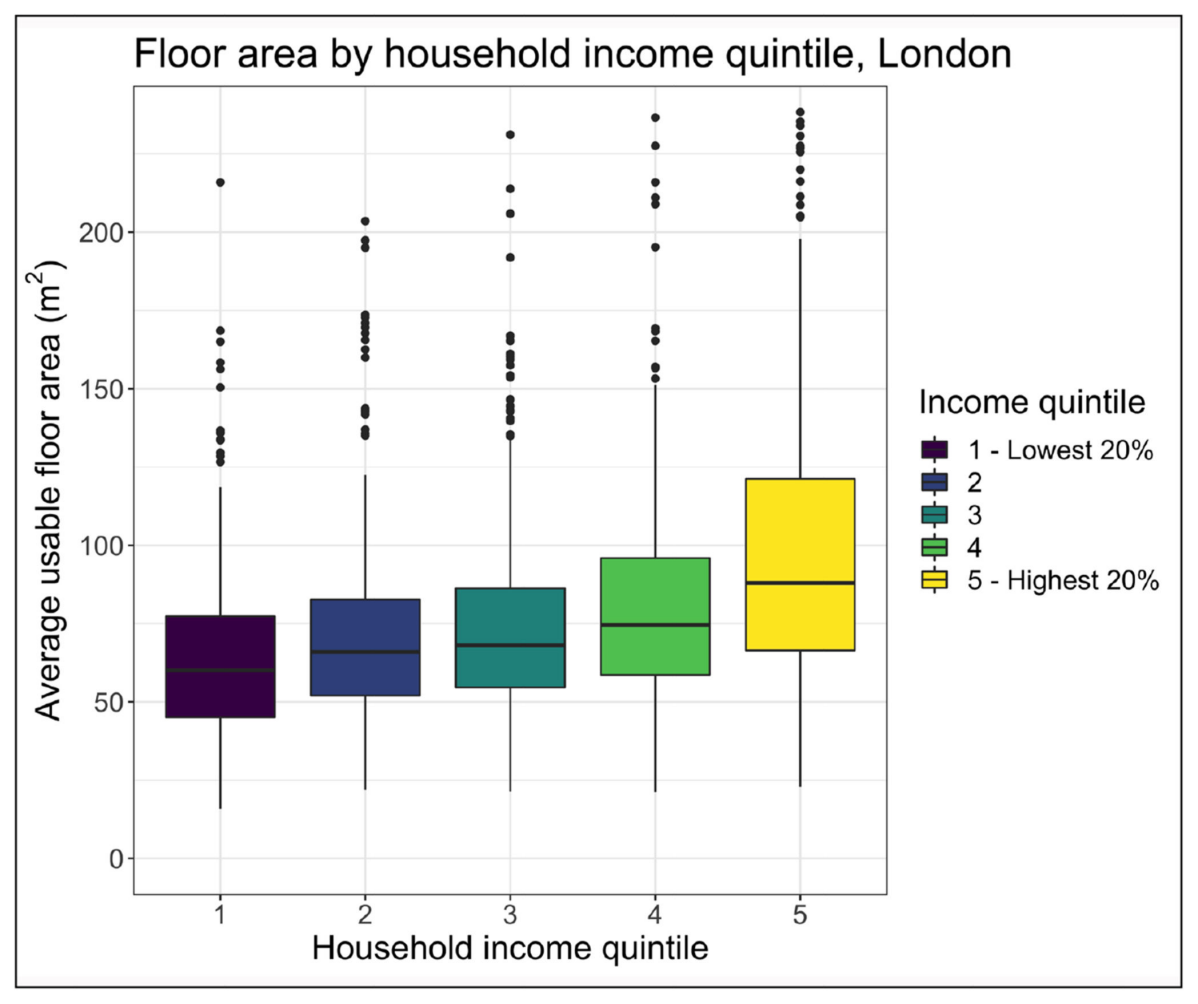
Average usable floor area by socioeconomic status (SES) group, London. *Source:* DCLG (2011).

**Figure 4:**
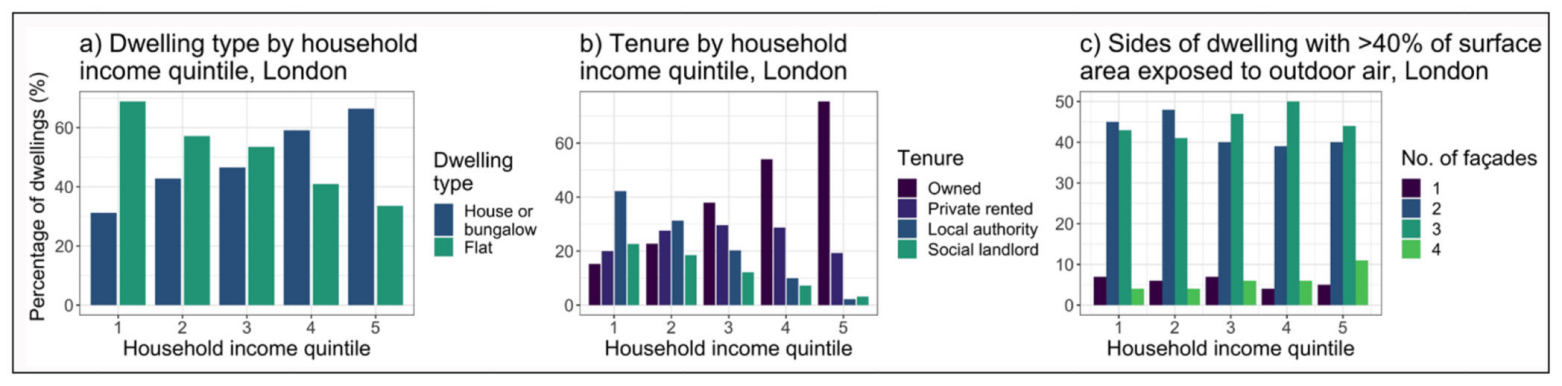
**(a)** Dwelling type; **(b)** tenure; and **(c)** number of exposed facades by socioeconomic status (SES) group, London. *Source:* DCLG (2011).

An analysis of dwelling geometry and tenure for the 2010–11 EHS dwellings (DCLG 2011) in London by household income quintile is presented in Figures 3 and 4. The data indicate that low SES groups more commonly live in flats and smaller dwellings, have fewer external facades to their dwellings, and hold a less secure tenure. The reduced number of facades may reduce the infiltration of outdoor-sourced air pollution, providing a degree of protection from high outdoor concentrations, but it results in negative implications for removing pollution from indoor sources (Shrubsole *et al*. 2016; Taylor *et al*. 2014).

#### 2.2.1 Indoor pollution from outdoor sources

Air infiltration rates and the subsequent air pollution infiltration factors for around 1.6 million spatially referenced London dwellings have previously been estimated by Taylor *et al*. (2019) using a building physics metamodel. The metamodel uses as input housing data derived from the Energy Performance Certificate (EPC) database (DCLG 2017b), parameterised using the UK Standard Assessment Procedure for the energy rating of dwellings (SAP) (BRE 2009). The infiltration factors for NO_2_ (mean = 0.4, range = 0.3–0.6) and for PM_2.5_ (mean = 0.6, range = 0.5–0.7) predicted by Taylor *et al*. (2019) compare well with previous estimates of infiltration factors predicted by the INDAIR/EXPAIR modelling framework (Dimitroulopoulou *et al*. 2006) (for NO_2_ mean = 0.5, for PM_2.5_ mean = 0.6).

**Figure 5:**
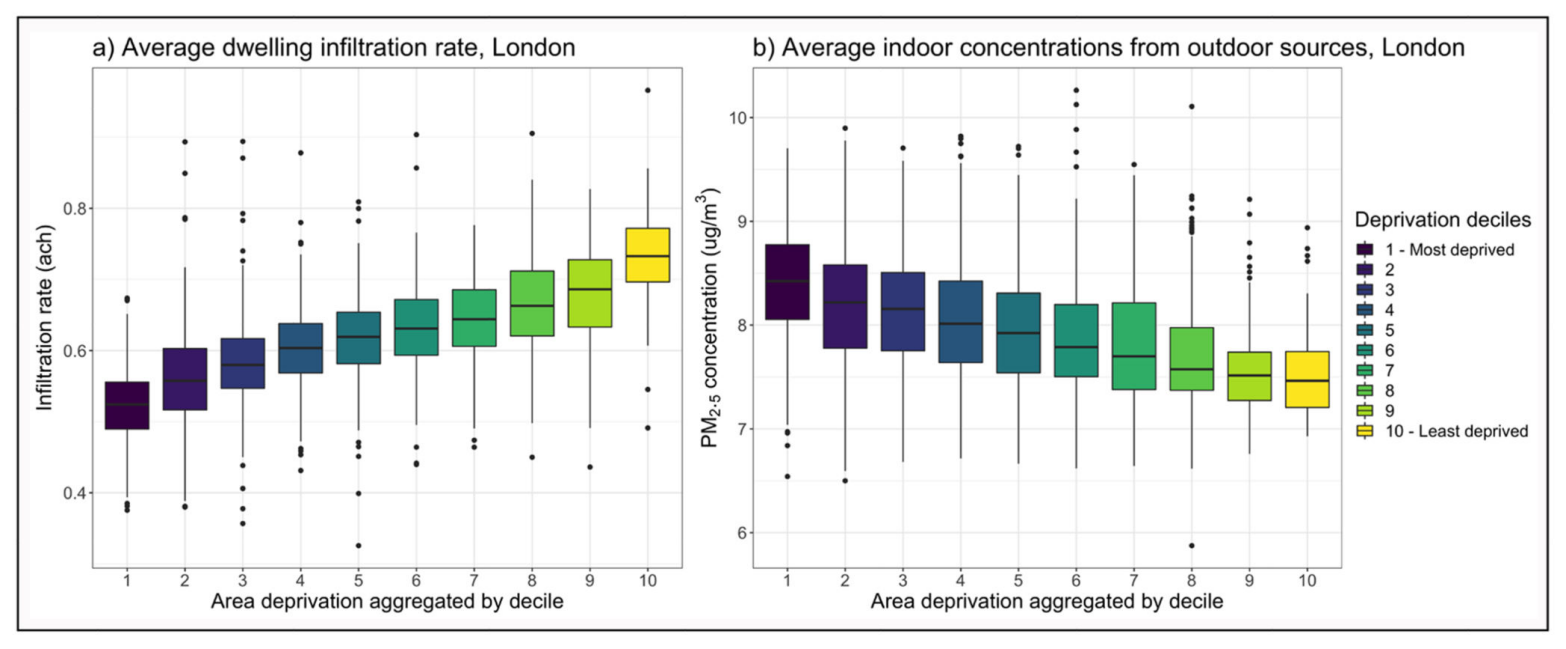
**(a)** Lower Super Output Area (LSOA) average of the estimated infiltration rates in dwellings by LSOA Indices of Multiple Deprivation (IMD); and **(b)** LSOA-average concentration of indoor PM_2.5_ from outdoor sources by LSOA IMD. *Note:* Annual average infiltration rates (a) are derived from the Energy Performance Certificate (EPC) database (DCLG 2017b), and infiltration factors used to determine indoor concentrations (b) were estimated using an existing metamodel by the authors (Taylor *et al*. 2019), developed using EPC data (DCLG 2017b).

To illustrate how infiltration rate varies by SES status in London, individual dwelling infiltration data were aggregated by LSOA and compared against IMD deciles. Results indicate that dwellings in higher income deciles are generally less airtight that those in lower IMD deciles (Figure 5(a)), reflecting the conclusions of Shrubsole *et al*. (2016). In addition to the physical characteristics of the dwellings (Figures 3 and 4), this may be in part due to social housing, which has a higher average energy efficiency than privately owned or rented housing stocks, likely due to the housing stock being newer and local council investment in energy-efficiency retrofits (Hamilton *et al*. 2014).

This increased airtightness can limit the infiltration of outdoor air pollution into the indoor environment, helping mitigate exposure to outdoor air pollution whilst indoors. To examine this, modelled infiltration factors for individual dwellings were spatially joined to the modelled outdoor air pollution grid used to derive Figure 1, and then used to estimate the average amount of outdoor air pollution infiltrating indoors for each LSOA. The relationship between modelled average indoor PM_2.5_ from outdoor sources and IMD is shown in Figure 5(b). This indicates that the lower average infiltration rates in low SES dwellings are not sufficient to offset disparities in outdoor air pollution levels, and that lower income households continue to experience higher levels of indoor air pollution from outdoor sources.

#### 2.2.2 Indoor pollution from indoor sources

Indoor sources of NO_2_ and PM_2.5_ include those from indoor appliances such as gas cookers and heating systems, as well as occupant-specific behavioural factors such as smoking and different cooking styles (Abdullahi *et al*. 2013; Hill *et al*. 2001). Increased airtightness in naturally ventilated dwellings leads to low background air-change rates. This can mean a reduced ability to remove indoor-generated pollutants if no additional purpose-provided ventilation is employed while pollution is being generated, *e.g*. extract ventilation. The increased airtightness of low-income housing in London shown above risks increasing concentrations of indoor-sourced air pollution (Shrubsole *et al*. 2016).

The physical layout of buildings can also impact indoor air pollution concentrations. Given an indoor source emitting air pollutants at a certain rate, a smaller volume will reach higher concentrations faster than the equivalent source for a larger volume space in the absence of additional ventilation.

Figure 3 shows a stark contrast in housing sizes between different income groups in London. Issues with low background air-change rates and dwelling volume can be mitigated by having adequate purpose-provided ventilation in dwellings. However, there are also differences in the operation of purpose-provided ventilation across socioeconomic groups. The EHS indicates that action is required to repair extractor fans in the lowest quintile income group in 57.5% of houses versus only 7.7% in the highest quintile income groups (DCLG 2011). Similarly, the EHS surveyors’ subjective assessment of the risk of damp and mould is higher in the lowest quintile income groups (29.1%) than in the highest (7.7%).

**Figure 6:**
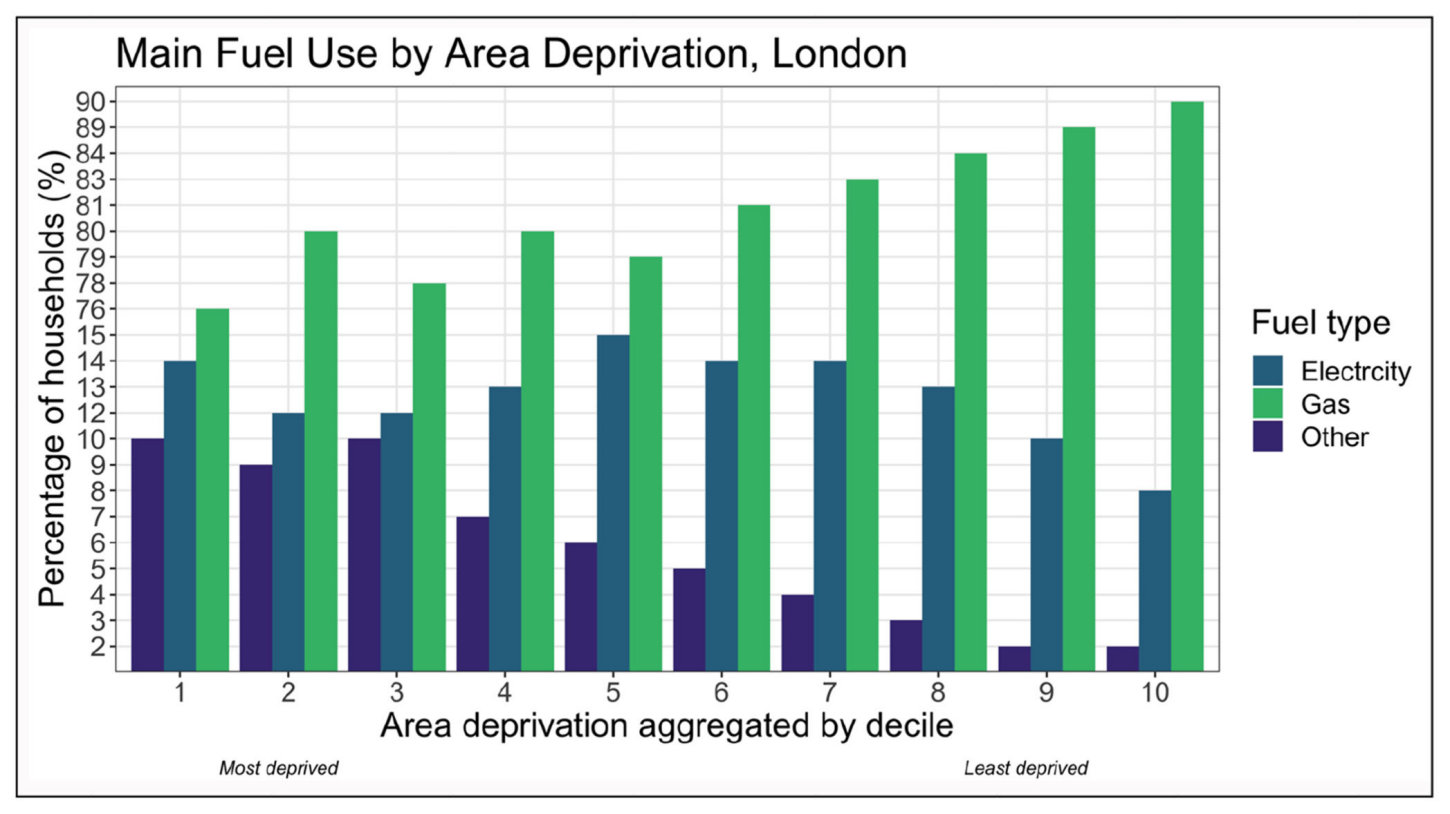
Proportion of households, per Indices of Multiple Deprivation (IMD) decile, using electricity, gas or other fuel types across London. *Source:* Data are from the Energy Performance Certificates (EPC) (DCLG 2017b).

The analysis of the EHS and EPC shows little evidence to indicate significant variations in indoor heating or cooking amenities that rely on polluting fuels—such as solid fuels—across socioeconomic groups in London (Figure 6). On the contrary, highest quintile income groups are more likely to use gas as their main fuel (91.7%) than lower income groups (86.4%), and more likely to have an open solid-fuel fire in their homes for secondary heating (20.6% versus 12.7%). However, the efficiency of heating and cooking systems may vary between groups. According to the Carbon Monoxide Cross Government Group report (HSE 2019), when smoke or fire were excluded as the source of carbon monoxide (CO), the highest proportion of unintentional exposures in 2018/19 was caused by domestic boiler issues (29.9%), cookers (7.1%) and domestic wood/coal-fire burners (4.1%). Low SES households may be disproportionately affected (Roca-Barceló *et al*. 2020) because they may be less able to properly maintain or repair inefficient or broken systems due cost, and since the majority of low-income households rent from private or social landlords (Figure 4(b)) they are reliant on someone else to organise and pay for repairs.

**Figure 7:**
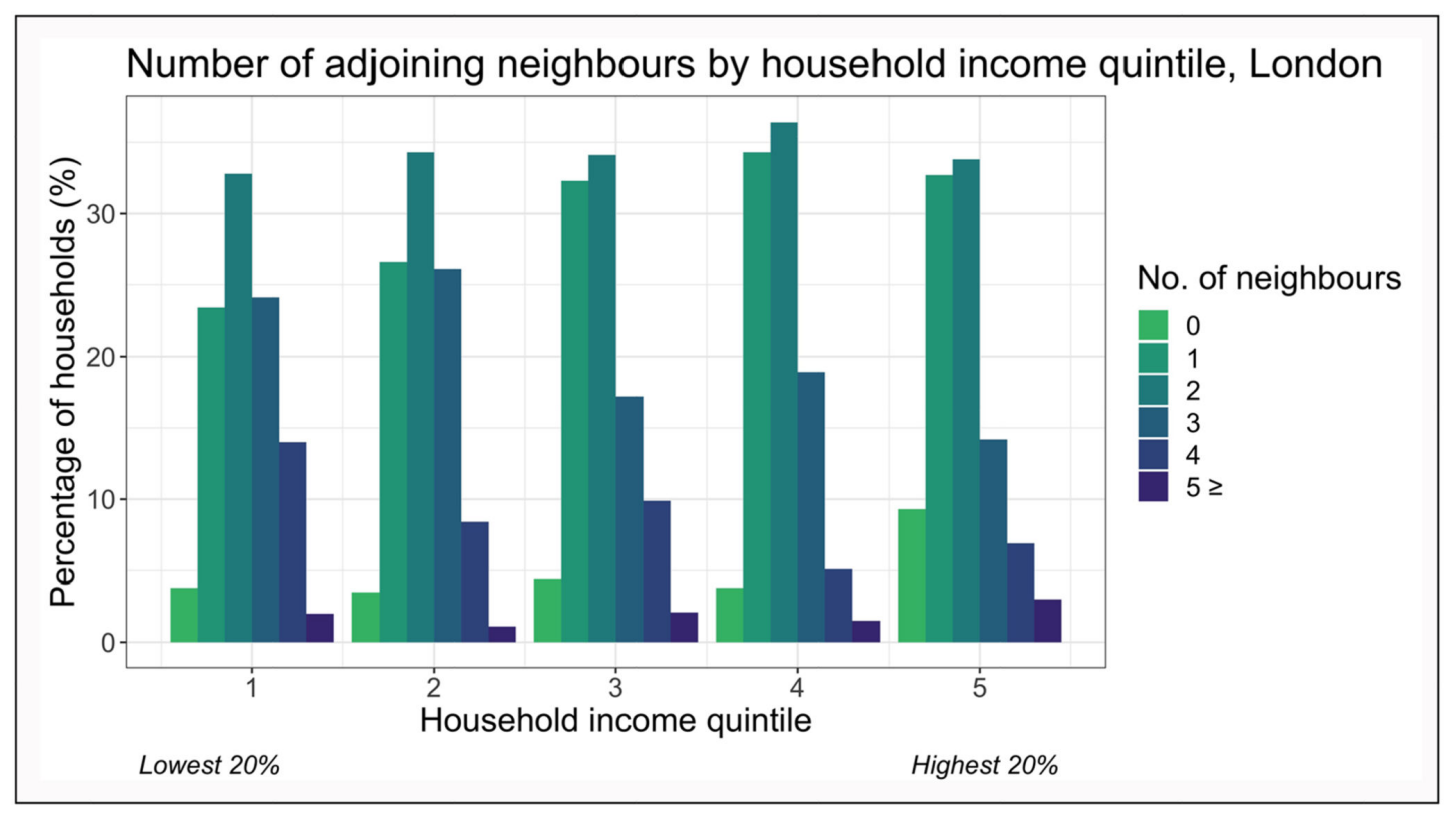
Percentage of households by income quintile that have adjoining neighbours. Mid-floor flats are assumed to have adjoining neighbours above and below. *Source:* Data are from the English Housing Survey (EHS) (DCLG 2011).

Low SES households are also more likely to live in flats than houses (Figure 4(a)), with party walls and/or floors separating dwellings. High-density housing increases the likelihood of air pollutants from adjoining dwellings or commercial premises entering the dwelling. Modelling studies have shown the ability of pollutants to travel between flats in multi-unit buildings (Ai *et al*. 2013; Mu *et al*. 2016; Zhou & Deng 2014). Figure 7 shows an analysis of London dwellings in the EHS, indicating that lower income households have a greater number of walls, ceilings and floors that adjoin neighbouring dwellings than higher income households, leading to a greater risk of pollution from neighbouring dwellings entering the household. In dwellings that share a building with a commercial premises, low-income households have a higher likelihood that the business involved is in food services (29%) than non-low-income households (23.6%), with implications for the infiltration of air pollution from commercial kitchens (DCLG 2011). Concentrations of indoor pollution from indoor sources will therefore also depend on the amenities in neighbouring dwellings as well as behaviours (discussed in Section 2.3). Airtightening of external building surfaces during energy-efficient retrofits risks increasing the infiltration of environmental tobacco smoke (ETS) between dwellings in multifamily units (Fabian *et al*. 2016).

### 2.3 Low SES Communities Have Behaviours That Can Aggravate Indoor Concentrations

Occupant behaviour is a similarly critical factor in determining indoor air pollution. The extent of pollution-generating activities (such as smoking and cooking), as well as the actions occupants take to ventilate their housing, can be significant drivers of indoor concentrations and can vary across socioeconomic groups.

Stark disparities exist in the underlying smoking rates between socioeconomic groups. In 2018, 25.5% of those working in the UK in routine and manual occupations smoked compared with 15.7% of those in intermediate occupations and 10.2% of those in managerial and professional occupations (ONS 2019a). Though households with smokers have decreased following national smoking bans (Mons *et al*. 2013), a survey reports that 13% of people continue to smoke inside their home on most days, whilst 21% reported exposure in their home from someone smoking elsewhere, such as a neighbour (ASH 2018). Various studies have shown that in homes with smokers resident, low-income homes have higher levels of ETS (Ferguson *et al*. 2020). Even in the absence of indoor smoking, second-hand smoke can be introduced into a dwelling through shared hallways or adjacent apartments from smoking to non-smoking units in multifamily complexes (Fabian *et al*. 2016; Kim *et al*. 2017). A simple probability calculation using data from Figure 7 indicates that if indoor smoking rates are assumed to be 13% across the population, low-income households have a 4% greater chance of living next door to at least one smoker than a higher income household in London solely due to the greater numbers of adjoining neighbours.

Cooking practices may also vary across socioeconomic groups. Adams & White (2015) found that those from the least affluent group spent between 10 and 20 minutes longer cooking per day than the other groups. Longer cooking times are associated with higher concentrations of indoor air pollution and levels can also vary according to the cooking techniques used, where barbequing and sautéing techniques have higher peak concentrations of PM than frying and oven cooking (Abdullahi *et al*. 2013).

Occupant ventilation behaviours may also vary between income groups. Security concerns may arise from living in low-income areas, lowering rates of window opening, reducing ventilation and the removal of pollutants from indoor sources; 70% of respondents in London reported only opening one or no windows at night due to security risks in a survey on occupant behaviour (Mavrogianni *et al*. 2017). Window-opening behaviours between households of different SES have been suggested as a driver of inequalities in CO poisoning (Roca-Barceló *et al*. 2020).

To illustrate the potential influence of longer cooking duration, smoking, dwelling type and working extract ventilation on indoor air pollution levels, indoor air pollution was modelled using EnergyPlus for a typical year (it is described in detail in Appendix 1 in the supplemental data online). EnergyPlus is a whole-building simulation tool that models building performance—including indoor air pollution levels—using building characteristics such as geometry, building materials, floor space and occupant behaviour (*e.g*. window-opening frequencies) as inputs (US DOE 2020). Table 2 illustrates the modelled daily mean, minimum and maximum PM_2.5_ concentrations in two dwelling types with different indoor source scenarios. Two dwelling types (detached and a high-rise flat) were considered due to their contrasting average floor areas (173 and 60 m^2^, respectively) and rates of inhabitation by low-income households (14% and 36% of households in London below 60% of the median household income, respectively) (DCLG 2011). A London average for each dwelling type was taken for building permeabilities (Taylor *et al*. 2019). The results indicate that small variations in cooking duration can have substantial impacts on the peak and mean indoor air pollution levels, with the effects worse in the smaller high-rise flat compared with the larger detached home due to the smaller internal volume. Cooking without an extractor fan increases indoor concentrations substantially, particularly in the smaller high-rise flat. Data are in line with empirical work, which has indicated that cooking activities are a significant contributor to indoor levels of air pollution (Jones *et al*. 2000; Wan *et al*. 2011). The results illustrate how some of the issues prevalent in low-income households—longer cooking periods, non-functioning extract ventilation and smaller floor areas—can act to substantially increase indoor air pollution levels.

### 2.4 Low SES Households Spend More Time at Home

In addition to indoor air pollution concentrations, the extent to which populations are exposed to indoor air pollution in the domestic environment will be determined by the amount of time they spend at home (Dimitroulopoulou *et al*. 2006; Milner *et al*. 2011). In England, 25% of those living in the most deprived neighbourhoods are unemployed compared with just 2% of those in the least deprived neighbourhoods (ONS 2019b). Unemployment is a significant predictor of more time spent at home (Krueger & Mueller 2012). Additionally, security concerns may arise from living in low-income areas, deterring people from leaving their home. An analysis of qualitative case-study interviews in an area of North London found that local residents would restrict the extent of their spatial and temporal movement outside of the home if they had perceptions of high rates of neighbourhood crime (Whitley & Prince 2005). This phenomenon was particularly pronounced in low-income residents and the authors termed these behavioural impacts as *time-space inequalities*.

These findings are supported by research that suggested children from low-income families are more likely to spend time indoors during out-of-school hours as a result of a lack of after-school club opportunities and low parental perceptions of the surrounding area (Eyre *et al*. 2014). Low-income homes are more likely to be equipped with media devices, such as televisions and video games, than outdoor sport equipment, further facilitating sedentary childhood behaviour (Tandon *et al*. 2012).

It is not just the duration of time at home but also the numbers of individuals at home. Low-income households are more likely to be overcrowded or have high levels of occupant density, which are strongly linked to poorer IAQ (Brown *et al*. 2015). This may be due to particle resuspension arising from occupant movement (Klepeis & Nazaroff 2006) and higher frequencies of pollution-generating activities, such as longer cooking durations to accommodate a larger household (Singer *et al*. 2017).

**Figure 8:**
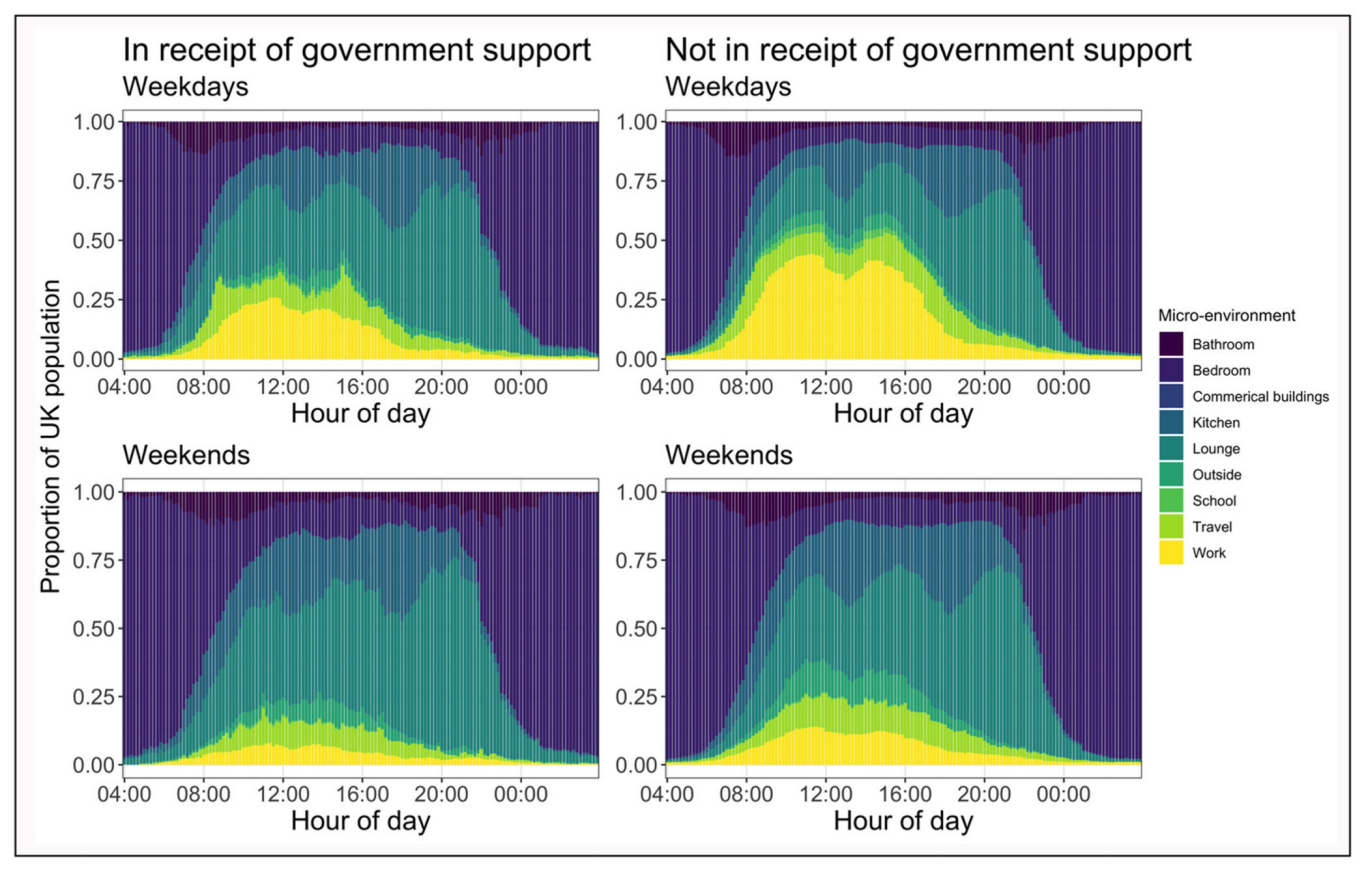
Time activity patterns on weekdays and weekends for different socioeconomic groups within the UK. Plots show the proportion of each population being in one of 9micro-environments, over 10-minute intervals.

Differences in time spent at home are illustrated by analysing the 2015 UK Time Use Survey (Gershuny & Sullivan 2017). Figure 8 shows the location of surveyed individuals at different times of the day. The socioeconomic indicator chosen for analysis was whether or not the household was in receipt of at least one of the following: unemployment-related benefits, income support, universal credit, disability benefits or tax credits. Summary statistics of the time spent at home for each socioeconomic group are outlined in Table 3. It illustrates how those in low SES groups spend a greater amount of time at home relative to the wider population, and emphasises the importance of the indoor environment for air pollution exposures. In addition to low-income populations, children, retired people and homemakers are all recognised as subgroups of the population who may spend more time in the home (Schweizer *et al*. 2007). Acknowledgement of this has led to research examining indoor air pollution exposure in older populations across Europe (Bentayeb *et al*. 2013, 2015; Mendes *et al*. 2016) and the role of IAQ in childhood health is of growing interest in the UK (Holgate *et al*. 2021).

### 2.5 Underlying Health Issues

In the UK, respiratory and cardiovascular diseases are two of the health conditions primarily responsible for the growing divide in health inequalities between the most and least deprived areas of the country observed over the past two decades (Bennett *et al*. 2018). The prevalence of such diseases is typically higher in areas of lower SES than their more affluent counterparts (Bennett *et al*. 2018), which in turn exacerbates the effects of exposure to air pollution (Forastiere *et al*. 2007).

Several wider determinants of population health can lead to health inequalities between SES groups. In the case of air pollution, population susceptibility is influenced by underlying health conditions, material deprivation and psychological stress. Examples of material deprivation include access to healthcare or a poor diet, in which the latter is a factor known to influence susceptibility to the negative effects of air pollution and is strongly linked to income class (Stamatakis *et al*. 2010). Lack of physical activity is another risk factor for poor underlying health. In addition to proximity to traffic, economically deprived areas have less access to good-quality greenspace, meaning the communities at greatest risk of poor physical and mental health often have limited opportunity to use such environments for exercise (PHE 2020).

This results in a triple jeopardy scenario whereby not only are low-income groups subject to disproportionate levels of indoor air pollution, but also their underlying health conditions mean they suffer to a greater extent upon exposure, compared with individuals without pre-existing conditions. As health issues worsen, individuals may be forced to spend more time at home, shifting the balance of air pollution exposures increasingly towards the indoor domestic environment.

## 3 Systemic Inequalities

Indoor air pollution exposures are due to a set of complex and often interrelated variables relating to occupant behaviour, housing and appliance characteristics, and community and the wider social and physical environment. Several factors that influence exposure to indoor air pollution are discussed, and evidence is shown that many of these factors may lead to unequal exposures to indoor air pollution across socioeconomic groups in London.

**Figure 9:**
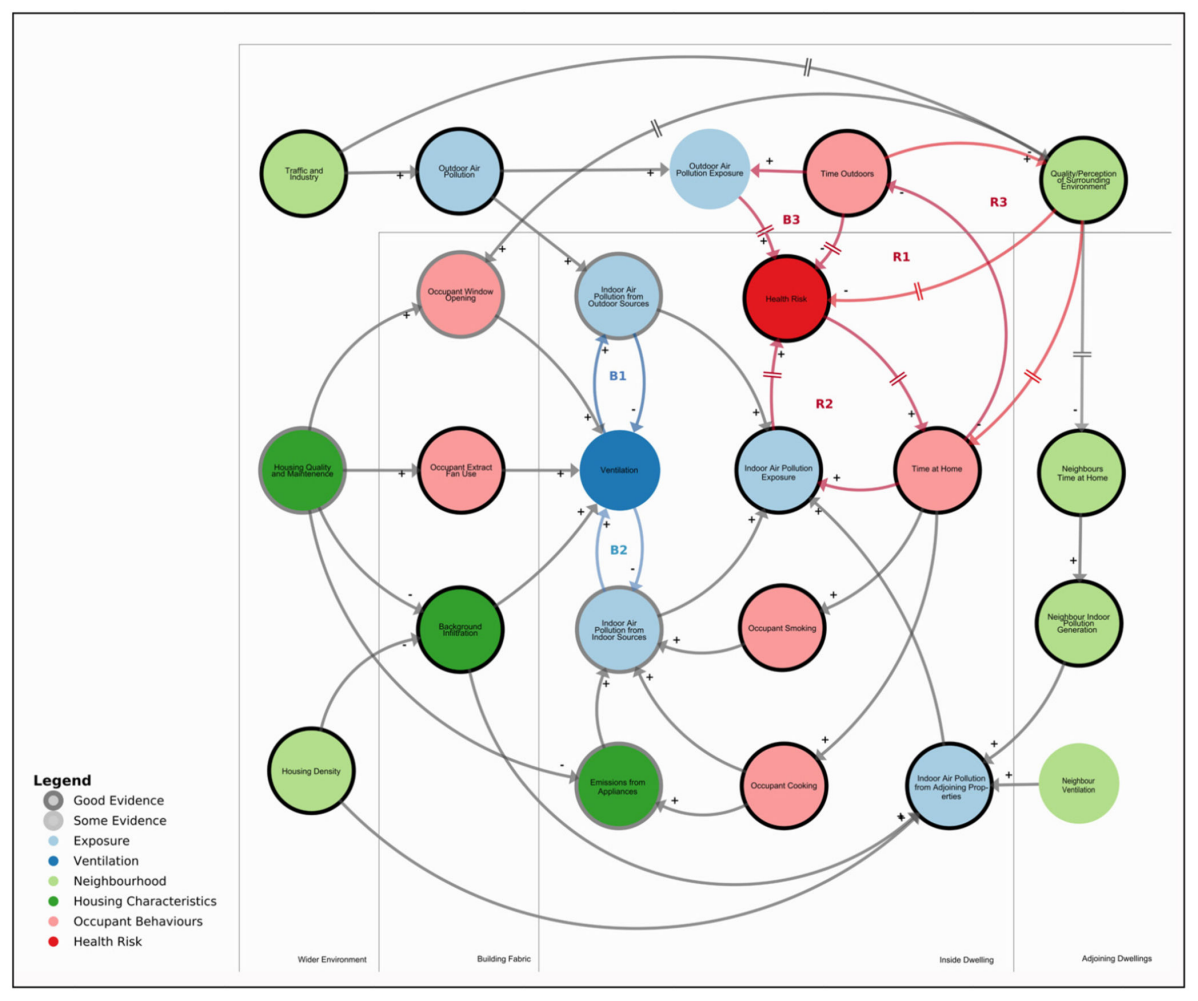
Systems diagram of the factors influencing indoor air quality (IAQ) in dwellings. *Note:* The boundary of each element indicates a qualitative description of the degree of evidence for socioeconomic disparities described in the paper. For the different components, see the Appendix in the supplemental data online. For an interactive version of the systems diagram, see https://kumu.io/jonathontaylor/indoor-air-pollution#systemic-inequalities.

Systems thinking is an approach that can highlight interactions and feedbacks across factors within complex systems such as indoor air pollution exposures and health. Figure 9 presents an initial systems diagram of indoor air pollution exposure, highlighting the feedback relationships between various factors within the system, and the availability of evidence and evidence gaps. This model was derived from the above review, and connections between the elements—which show positive or negative relationships—and are derived from indoor and urban air quality modelling theory. Arrows and links represent causal relationships in the systems diagrams, often referred to as causal loop diagrams. Here, positive a positive relationship indicates a relationship goes to the same direction and is represented by a plus polarity: ‘+’. A negative relationship indicates an inverse relationship and is represented by a minus polarity: ‘−’. Links with a ‘||’ symbol indicate a delay in the response.

Elements in the system are coloured according to whether they are related to occupant behaviours, housing, neighbours or the surrounding neighbourhood. Where there is evidence of poorer conditions for lower socioeconomic groups based on the above review, from London or elsewhere, a black boundary is added. Where there is less evidence, but it has been suggested that disparities may exist, a grey boundary is added. To reduce the complexity of the model, the system is bounded by the immediate local environment and excludes wider societal issues. For a focused breakdown of the diagram, referring to each subsection of the review, see Appendix 2 in the supplemental data online.

At the core of the diagram is a series of feedback loops. These loops are either balancing loops—which act as negative feedbacks, countering changes in the system with an effect to reverse the change—or reinforcing loops, which act as positive feedbacks, compounding system changes by increasing the change:

- Balancing loops (*B1* and *B2*—shown in blue) are related to ventilation. *B1* illustrates how increases in ventilation can increase indoor levels of air pollution from outdoor sources. As indoor concentrations of outdoor pollution increase, occupants may then act to reduce ventilation. *B2* shows how increases of indoor air pollution from indoor sources can lead occupants to increase ventilation, which decreases indoor air pollution from indoor pollutants. The loops act in conflict with each other, illustrating how, in areas of high outdoor air pollution, residents may face the ventilation dilemma of either elevated indoor exposures to indoor or outdoor-sourced pollution.
- Four loops (*B3, R1, R2 and R3*—shown in red) are related to pollution exposures, behaviour and health risks. The balancing loop *B3* (see Figure S5a in Appendix 1 in the supplemental data online) illustrates how increased exposures to outdoor air pollution can gradually lead to increased health issues, resulting in individuals spending more time at home and a consequent reduction in outdoor air pollution exposures. These are in contrast to reinforcing loop *R1* (see Figure S5b online) that shows how an increased time at home may decrease the time spent outdoors, gradually increasing health risks due to, for example, a lack of physical activity, which further increases time at home. Reinforcing loop *R2* (see Figure S5c online) illustrates how increased time at home due to air pollution-related health problems reinforces exposures to indoor air pollution. Therefore, as health issues due to indoor and outdoor air pollution increase, the exposure balance shifts towards the indoors; this is likely accelerated by the higher rates of underlying health issues in low-SES populations. Reinforcing loop *R3* (see Figure S5d online) describes how as individuals’ perception of their local environment decreases, they gradually spend more time indoors, further reducing their perception of their local environment.

The systems diagram also shows how external inputs to the balancing loops can help drive inequalities in indoor air pollution exposures. It illustrates how low SES households have relatively little opportunity to improve their IAQ other than changing their own behaviours, and in many cases these behaviours are determined or restricted by wider systemic issues such as the local environment and the quality or maintenance of the dwelling. Even with behavioural changes, low SES occupants may continue to be exposed to greater levels of indoor air pollution than higher income groups due to factors outside their direct control such as dwelling location and neighbourhood behaviours. In contrast, higher SES individuals will have a freedom of choice where poor environmental quality can be offset by compensatory benefits of central living, while such benefits may not be available to economically constrained households (Walker *et al*. 2003).

The systems diagram highlights the self-reinforcing inertia of a system where low SES is connected to high levels of exposure to poor air quality. If interventions do not address these underlying systemic inequalities, then it is highly likely that lower income individuals would continue to be exposed to elevated levels of indoor air pollution. The diagram represents an initial analysis, supported by evidence from the review, and further work is necessary to extend and validate the model, which may be done, for example, in stakeholder workshops. It has been developed for the UK context with predominantly mixed-mode-ventilated dwellings, and there are opportunities to adapt it for areas where mechanical ventilation systems are more common.

## 4 Discussion and Potential Interventions

Previous work has reviewed evidence on how concentrations of indoor domestic air pollution may vary between SES groups (Ferguson *et al*. 2020). The work carried out in this paper explores the driving factors for why low-income individuals may be disproportionately impacted by household air pollution. The focus here has been on collecting evidence, modelling and analysing data from London, but these factors are likely drivers for exposure inequalities in other areas around the world.

This paper has focused on air pollution in dwellings only. Other indoor environments will be significant drivers of population exposure, but were not discussed in the work carried out here. One important environment is likely to be schools, given the vulnerability of children to indoor air pollution. Research over recent years has found that IAQ in London’s school’s breach World Health Organization (WHO) guidelines and exceed ambient outdoor levels (Mumovic *et al*. 2018) in some areas of the city. Of London’s 1777 primary schools, 433 are located in areas where local NO_2_ levels exceeded the EU limits, 82% of which are in deprived areas (Brook & King 2017). Further research could examine disparities in time-weighted air pollution exposure between SES groups and various indoor and outdoor environments. In addition, the present study has focused on pollutants that have shown evidence of higher levels in low SES households; other indoor pollutants such as radon and volatile organic compounds (VOCs) may actually be lower in less affluent households (Ferguson *et al*. 2020).

### 4.1 Potential Interventions

**Figure 10:**
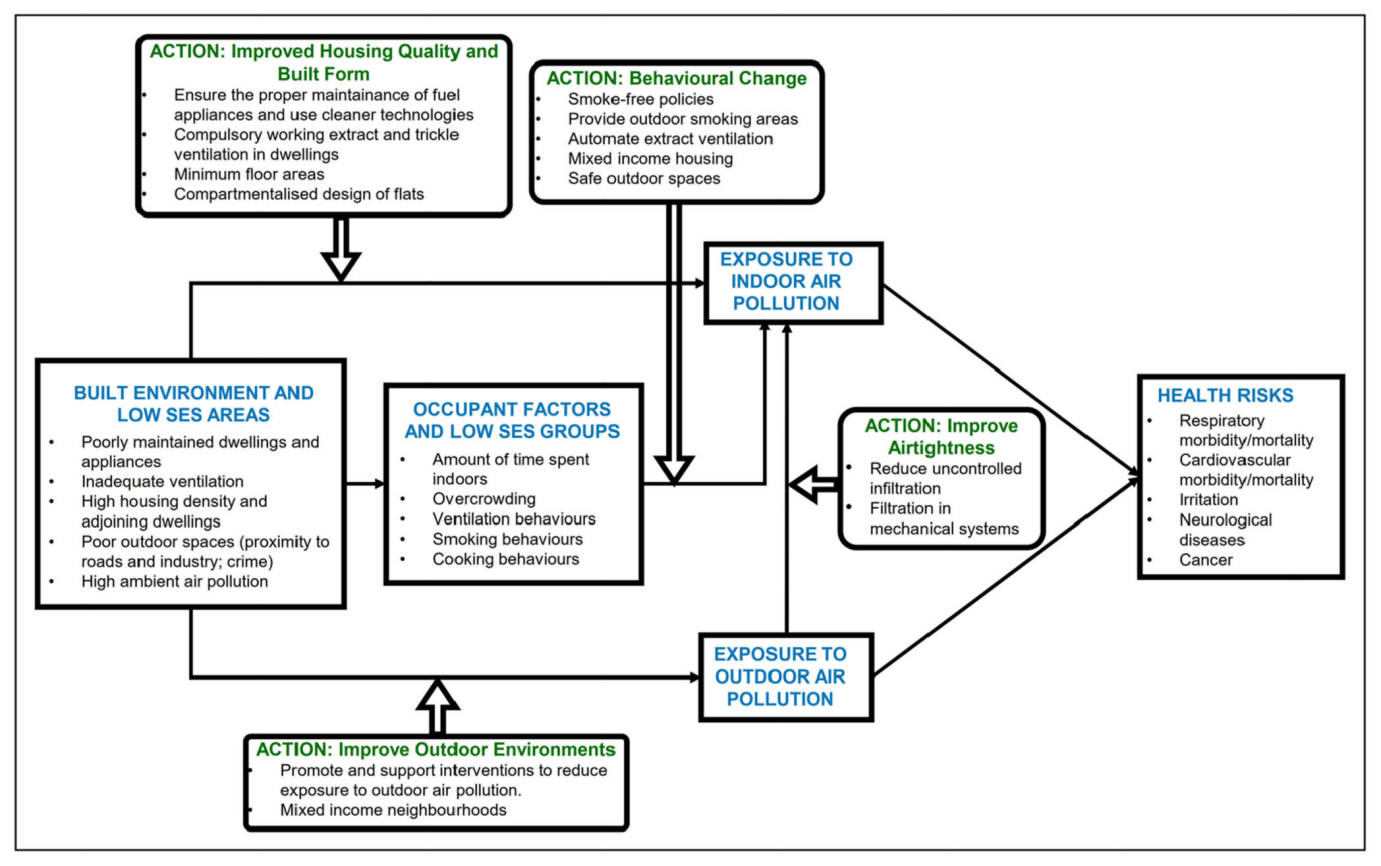
System approach for indoor air pollution disparities between socioeconomic groups in the UK.

Health inequalities can be addressed through the introduction of public health initiatives that target health disparities from both social and economic inroads. Potential systemic interventions to reduce indoor air pollution exposures are shown in Figure 10 and discussed below.

#### 4.1.1 Improving outdoor environments

The data suggest that low SES individuals tend to spend more time indoors. Improving access to local high-quality, safe and low-traffic outdoor spaces may act (1) to reduce outdoor air pollution levels and its subsequent infiltration indoors; (2) encourage residents to spend less time indoors and more time outdoors, which may lead to an increase in physical activity and reduction in underlying health issues; and (3) potentially lead to more use of natural ventilation to remove indoor-generated pollutants. However, unless outdoor concentrations are significantly reduced,spending more time outdoors may not lead to a reduction in absolute levels of air pollution exposure but rather a switch in exposure environments.

There has been substantial work to reduce air pollution levels in London as part of the Mayor’s Environment Strategy (GLA 2018). Annual average outdoor concentrations have decreased from 50 to 39 μg/m^3^ for NO_2_ and from 13 to 11.6 μg/m^3^ for PM_2.5_ between 2016 and 2019 (GLA 2020a). As a major source of London’s outdoor air pollution, resources have been directed towards managing the capitals vehicle fleet. The Ultra-Low Emission Zone (ULEZ) was introduced in April 2019 and has been attributed a 36% decrease in roadside NO_2_ emissions from transport (GLA 2019). Vehicles driving in areas of Central London operating under ULEZ regulations must meet a given emission standard or incur a fee to travel within the area. Similar measures have been placed on buses, with the city committed to phasing out diesel buses by 2037 in order to bring the city’s air pollution concentrations within acceptable limits. Low-traffic neighbourhoods may help to reduce exposure inequities, as low-income households are more likely to live within them in London (Aldred *et al*. 2021).

Investments to reduce outdoor air pollution and traffic, and improve local green space, safety and local amenities can encourage residents to spend more time outdoors and reduce indoor pollution levels. Inadequate access to green infrastructure has been identified as a driver of health inequalities between socioeconomically and ethnically diverse populations in England (Roe *et al*. 2016). Investing in infrastructure to encourage walking increases physical activity across different age and socioeconomic groups (PHE 2017) and creating compact neighbourhoods that facilitate physical activity is one of the key principles underpinning the NHS’s Healthy New Towns Programme (NHS 2019).

Though these actions demand direct engagement with residents and collaboration efforts from the planning, housing, transportation, health sectors and academic research, such efforts may act to counter not only exposure to poor IAQ but also other housing-related inequalities. Persistently poor neighbourhoods often struggle with disinvestment, crime, a lack of political power, and poor physical and green infrastructure. Whilst they have faced criticism (Darcy 2010), mixed-income communities—which include a proportion of affordable housing—are a policy measure aimed at improving such neighbourhoods by promoting more cohesive societies via the diversification of tenure and housing types (Tunstall & Lupton 2010). As the underlying social, economic and environmental conditions will vary between locations, mixed-income housing and improving green space are examples of policies that must be implemented and tailored to the needs of the local environment, since suitable interventions will vary according to the local context.

#### 4.1.2 Improving housing quality and urban form

There is a recognised need to improve household energy efficiency in the UK (Hamilton *et al*. 2014). Increased energy efficiency would benefit fuel-poor households that struggle to afford to keep their homes sufficiently warm in winter. One way to achieve this is through reducing heat losses by increasing the airtightness of dwellings; this may also reduce infiltration of outdoor air pollution indoors (Shrubsole *et al*. 2016). However, more airtight building envelopes may have the unintended consequence of increasing exposure disparities between SES groups by reducing the rate of removal of indoor sourced air pollution (Shrubsole *et al*. 2016), and can increase pollutant transmission between flats in buildings. Therefore, energy-efficiency retrofits require sufficient compensatory ventilation, particularly in multi-dwelling buildings. Policies aimed at ensuring all housing has sufficient ventilation—including automated extractor fans—and that mechanical ventilation is maintained and operational would significantly reduce indoor air pollution exposures. Increased compartmentalisation between flats would help to reduce air pollution entering from adjoining dwellings.

Another means to improve IAQ is to remove indoor sources. While the analysis shows no apparent inequity in polluting fuel use across socioeconomic groups in London, providing clean fuels (such as hydrogen) and technologies for cooking and heating, and ensuring adequate maintenance of fuel appliances, is still essential.

In areas of high outdoor air pollution, high-efficiency particulate air (HEPA) filters in mechanical ventilation can be used as a means of removing particles infiltrating buildings, while portable air purifiers can be used to remove pollution from the indoor air (Cheek *et al*. 2020; Kelly & Fussell 2019). However, such systems may not necessarily lead to improvements in health outcomes (Brugge *et al*. 2017; Cheek *et al*. 2020; Padró-Martínez *et al*. 2015), and would incur purchase and running costs, likely making them inaccessible to low-income groups.

As with outdoor environments, mixed-income housing may help improve indoor air pollution equity by distributing the risks of air pollution exposure from neighbouring dwellings and ensuring residents have the resources and power to ensure the maintenance of the building and communal ventilation systems.

Data from the EHS estimate that there was a £19 billion backlog of repairs and maintenance in the social housing sector in 2000. A 50-year analysis of the EHS noted that though efforts had been made to improve social housing, 13% of homes in the social sector remained non-decent, and this figure was even higher for the private-rented sector, at 28% (DCLG 2017a). Therefore, national housing policies that require the improvement of social and privately rented properties will benefit low-income individuals who are more likely to occupy these tenures, and face challenges when trying to improve their property as repairs must be mediated through a third party. It is estimated that poor-quality housing costs the UK economy £2.5 billion per year (PHE 2017), therefore improving build quality can have far-reaching benefits by reducing costs and increasing the availability of resources across the healthcare system.

#### 4.1.3 Changing the behaviour of occupants

Behaviours in their own household is the only factor that low-income households may be reasonably expected to have the ability to change, but this can often be constrained by external factors: For example, occupants may wish to improve IAQ by opening windows, but be deterred due to the risk of neighbourhood crime (Mavrogianni *et al*. 2017). The provision of adequate ventilation has previously been addressed by building regulations in order to maintain IAQ, but there is a growing interest in how to approach changing population behaviour (Diepeveen *et al*. 2013). Policymakers should consider the degree to which policy intervention with public behaviour is deemed acceptable, which will critically affect the public resistance to the intervention and subsequent outcome.

Resources may be better directed towards national public awareness campaigns targeting populations at risk of elevated exposure to indoor air pollution. For example, the introduction of a smoking ban increased awareness of the hazards of indoor smoking (Jarvis *et al*. 2009). A review of indoor exposure to ETS across SES groups found exposure decreased over the duration of the study, with the authors suggesting this may be the effect of introducing national legislation in public spaces (Ferguson *et al*. 2020). Smoking in communal areas of flats was banned alongside the national 2007 smoking ban in England, but legislation does not apply to the individual units within the buildings. Given the evidence of indoor ETS ingress to adjacent dwellings, residents would benefit if policy were to incorporate private spaces in social housing in this framework. Such changes could be facilitated by the introduction of sheltered smoking units outside the premises. Smoke-free policies in low-income multifamily housing has shown promise in reducing second-hand smoke exposures in the US (Hollar *et al*. 2017).

This paper discussed how exposure to poor IAQ falls disproportionately on those of low SES. Many of the reviewed factors may also be important for other health-related exposures. Issues with fuel poverty and poor housing quality can lead to excessive exposure to indoor cold (Dear & McMichael 2011), and there is evidence to suggest that low-income communities are exposed to higher indoor temperatures during hot weather (Vellei *et al*. 2017). A report by the WHO (2019) on environmental health indicates increasing inequalities in energy poverty, thermal comfort, damp homes and noise perception across most countries in Europe, representing a common challenge. Exposure to noise pollution, which has similar outdoor disparities between income groups in London (Tonne *et al*. 2018), can be transmitted between dwellings in multifamily housing, and housing quality can impact the degree of transmission.

Given the amount of time spent at home, housing is an important environment in which unhealthy exposures and health inequalities may arise. Improving housing quality by reducing the risk of home environmental exposures improves population health and has wider reaching benefits of meeting the Sustainable Development Goals of reducing inequality and ensuring sustainable and equitable cities (DfID 2019). There are opportunities for policies to promote health and prevent disease by addressing the non-medical determinants of population heath (McGinnis *et al*. 2002), such as poor housing and environment. For indoor air pollution, policies that seek to reduce exposure inequalities by addressing the systematic factors described here may help to reduce health inequalities and the total air pollution-associated health burden.

## 5 Conclusions

In contrast to outdoor air pollution, indoor air pollution exposure disparities are under-researched. The evidence (data and analyses) described here provides exposure and behavioural data that will form the basis for future exposure modelling and health-impact analyses, and exemplifies how open government data can be used to evaluate the performance of the building stock and initiate progress and innovation within this sector.

The presented evidence demonstrates how systemic inequalities can cause exposure disparities of selected indoor air pollutants across SES groups. These disparities may then reinforce systemic inequalities via poorer health outcomes, shifting the balance of pollution exposure further towards the indoor environment. Given that these disparities will drive health inequalities, action to reduce exposure to indoor air pollutants for the most vulnerable is critical. This may include reducing outdoor pollution levels, a complete ban on indoor smoking, ensuring all extractor fans are properly functioning, and increasing public awareness around indoor air quality issues. Actions to reduce exposures are moving higher on the political agenda.

Further research is necessary to understand population exposure to indoor air pollution across socioeconomic groups, and the practical actions required to protect those from low-SES homes and address the growing health inequalities gap in the UK.

## Supplementary Material

S1

## Figures and Tables

**Table 1:** Outdoor air pollution exposure disparities in London.

REFERENCE	SOCIOECONOMIC INFORMATION	OUTDOOR EXPOSURE MEASURE	POLLUTANT	ASSOCIATION	SIGNIFICANCE
Pye *et al*. (2001)	UK Index of Deprivation	Outdoor concentration maps calculated using monitored data and dispersion coefficients from the UK emissions inventory	PM_10_ NO_2_	Air pollutant concentrations in Greater London increased with increasing levels of deprivation. This correlation was stronger for outdoor NO_2_ than PM_10_	PM_10_: ***p* = 0.01** NO_2_: ***p* = 0.01** ^d^
Goodman *et al*. (2011)	NDI^a^ HInc^b^ % in Employment EA^c^ From census data	Annual average NO_x_ levels were modelled with several models before using land use regression to predict concentrations on a 20 × 20 m grid	NO_x_	A 1 SD (standard deviation) increase was associated with a 1.6%, 1.1% and 1.5% increase in NDI score, income and employment, respectively, and a 2.2% *decrease* in educational attainment in NO_x_ concentration	All ***p* < 0.05**
Fecht *et al*. (2015)	Recipients of income support—The English Index of Deprivation	Spatial model overlaying with high-resolution air pollution maps with annual mean concentrations of PM_10_ and NO_2_	PM_10_ NO_2_	NO_2_ concentrations were 7.8 μg/m^3^ higher in the most deprived neighbourhoods than in the most affluent neighbourhoods	***p* < 0.05**
Tonne *et al*. (2018)	HInc—from the study questionnaire	Spatial exposure model that uses residential location, trips, mode of transport and time spent in non-residential locations between trips as inputs	PM_2.5_ NO_2_	Highest income group (> £75,000) had a lower residential NO_2_ level by 1.3 μg/m^3^ compared with the lowest (< £10,000). The equivalent difference in PM_2.5_ was 0.12 μg/m^3^	PM_2.5_: ***p* < 0.05** NO_2_: ***p* < 0.05**
Samoli *et al*. (2019)	Unemployment rate HInc Crimes per 100,000 inhabitants	Land-use regression model incorporating chemical transport modelling, land use and transport networks	NO_2_	Unemployment rate had a positive correlation coefficient = 0.381 with outdoor NO_2_ concentrations. Crimes per 100,000 inhabitants had a positive correlation coefficient = 0.850 with outdoor NO_2_ concentrations	Unemployment: ***p* < 0.05** Crimes: ***p* < 0.05**

a Neighbourhood deprivation index.

b Household income.

c Educational attainment.

d Values shown in bold are significant at *p* ≥ 0.05.

**Table 2:** Indoor PM_2.5_ concentrations throughout the week in the kitchen of a modelled detached and high-rise building with various indoor source scenarios.

INDOOR SOURCE	DWELLING ARCHETYPE					
	DETACHED(μg/m^3^)			HIGH-RISE FLAT(μg/m^3^)		
	MINIMUM	DAILY MEAN	MAXIMUM	MINIMUM	DAILY MEAN	MAXIMUM
No indoor sources	0.16	3.51	12.1	0.10	2.10	12.4
Baseline cooking duration	0.26	28.3	453.0	0.10	36.1	676.0
Baseline cooking duration without and extractor fan^a^	0.36	32.8	570.0	0.10	64.7	1,380.0
Baseline cooking plus smoking	0.52	41.8	477.0	0.13	53.5	804.0
+20 minutes of cooking	0.26	52.3	511.0	0.10	71.5	694.0

a Indoor concentrations were modelled with and without a working kitchen extractor fan. Minimums and maximums represent the lowest and highest concentrations in 10-minute intervals over the 365-day period.

**Table 3:** Summary statistics for the percentage of time spent at home by participant socioeconomic status (SES) and type of day for the UK survey population.

IN RECEIPT OF GOVERNMENT SUPPORT	DAY	*N*	MEAN (%)	SD (%)	MEDIAN (%)
Yes	Weekday	573	78.8	19.1	84.0
Yes	Weekend	566	82.9	20.4	89.6
No	Weekday	2,928	68.7	21.3	68.1
No	Weekend	2,997	78.3	20.1	83.3
