# Supplementary material for: Systemic inequalities in indoor air pollution exposure in London, UK
Source: Build Cities. Author manuscript; Available in PMC 2021 Jun 11. (PMC7610964; doi:10.5334/bc.100)
Supplement: S1 [file EMS124175-supplement-S1.pdf]

**Ferguson, L., Taylor, J., Zhou, K., Shrubsole, C., Symonds, P. & Davies, M. (2021). Systemic inequalities in indoor air pollution exposure in London, UK. *Buildings and Cities.***

# Supplemental Data

# APPENDIX 1

Simulations were run using EnergyPlus for using a weather file with typical conditions for London City. Building geometries are based on archetypes used in Taylor et al (2016), with permeabilities taken to be the average of each London dwelling type based on previous stock modelling Taylor et al (2019).

Indoor emission rates, deposition rates and production schedules are outlined below in Table S1. Outdoor $PM_{2.5}$ levels were kept at a static 13.3 μg/$m^3$ across all models, representing the London 2019 average ambient concentration (GLA, 2020). Smoking was assumed to occur eleven times per day, in line with ONS data which suggests current UK smokers consume an average of 11.3 cigarettes per day (ONS, 2019). Cooking schedules are based on those from Taylor et al (2016).

Extract fans, if present, were modelled to be used while cooking, with an extract rate according to building regulations (HM Government, 2013). No extra ventilation was modelled during smoking. Window opening was modelled when indoor temperatures exceeded 24C during the summer.

**Table S1.** Indoor emission rates, deposition rates and production schedules for $PM_{2.5}$.

| Source | Emission rate | Deposition rate | Schedule | |
|---|---|---|---|---|
| | | | ***Weekdays*** | ***Weekends*** |
| Baseline cooking | 1.6 mg/min[a] | $0.19h^{-1}$ [b] | 07:20 – 07:50<br>18:00 – 18:30 | 08:30 – 09:00<br>13:00 – 13:30<br>18:00 – 18:30 |
| +20 mins cooking | 1.6 mg/min[a] | $0.19h^{-1}$ [b] | 07:10 – 07:30<br>12:40 – 13:10<br>18:00 – 18:30 | 08:30 – 09:00<br>13:00 – 13:30<br>18:00 – 18:30 |
| Smoking | 0.9 mg/min[a] | $0.10h^{-1}$ [c] | 12:00 – 12:05 (kitchen)<br>12:30 – 12:35 (kitchen)<br>17:00 – 17:05 (kitchen)<br>18:00 – 18:05 (kitchen)<br>10:00 – 10:05 (living room)<br><br>11:00 – 11:05 (living room)<br>15:00 – 15:05 (living room)<br>16:00 – 16:05 (living room)<br>19:00 – 19:05 (living room)<br>20:00 – 20:05 (living room)<br>21:00 – 21:05 (living room) | |

*Sources:* [a] Dimitroulopoulou et al, 2006; [b] Long et al, 2001; [c] Klepeis et al, 2006.

## References

Dimitroulopoulou, C., Ashmore, M.R., Hill, M.T.R., Byrne, M.A., Kinnersley, R. (2006). INDAIR: A probabilistic model of indoor air pollution in UK homes. *Atmospheric Environment* 40, 6362–6379.

GLA – Greater London Authority. (2020). Air pollution monitoring data in London: 2016 to 2020. London.

HM Government (2013). Approved Document F - Ventilation. Building Regulations 2010.

Klepeis, N.E., Nazaroff, W.W. (2006). Modeling residential exposure to secondhand tobacco smoke. *Atmos. Environ.* 40, 4393–4407. https://doi.org/10.1016/j.atmosenv.2006.03.018

Long, C.M., Suh, H.H., Catalano, P.J. and Koutrakis, P. (2001). Using time-and size-resolved particulate data to quantify indoor penetration and deposition behavior. *Environmental Science & Technology*, 35(10), 2089-2099.

ONS – Office of National Statistics. (2019). Adult smoking habits in the UK: 2018. London.

Taylor, J., Davies, M., Mavrogianni, A., Shrubsole, C., Hamilton, I., Das, P., Jones, B., Oikonomou, E. and Biddulph, P. (2016). Mapping indoor overheating and air pollution risk modification across Great Britain: A modelling study. *Building and Environment*, 99, 1-12.

Taylor, J., Shrubsole, C., Symonds, P., Mackenzie, I. and Davies, M. (2019). Application of an indoor air pollution metamodel to a spatially-distributed housing stock. *Science of The Total Environment*, 667, 390-399.

# APPENDIX 2

This appendix shows the formation of the systems diagram.

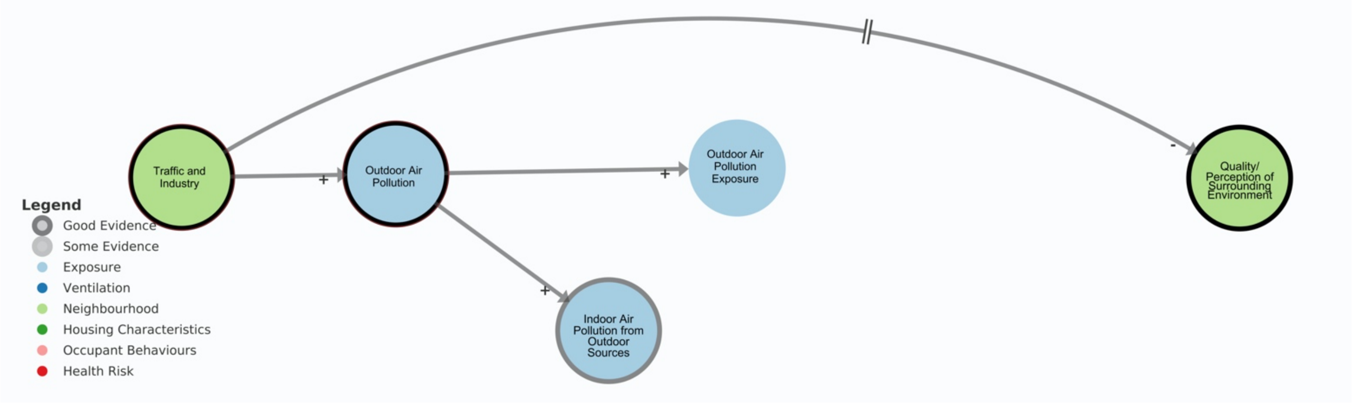


**Figure S1.** The relationship between traffic and industry and outdoor air pollution, described in Section 2.1.

Variables with black outlines are those which have empirical evidence to support socioeconomic differences in levels in London. Here, increases in the amount of traffic and industry will lead to increases in outdoor air pollution and a gradually reduction in the quality and perceived quality of the surrounding environment. There will be a more immediate increase in outdoor air pollution. Summarised evidence supports socioeconomic disparities in traffic, outdoor air pollution, and perceived quality of the outdoor environment.

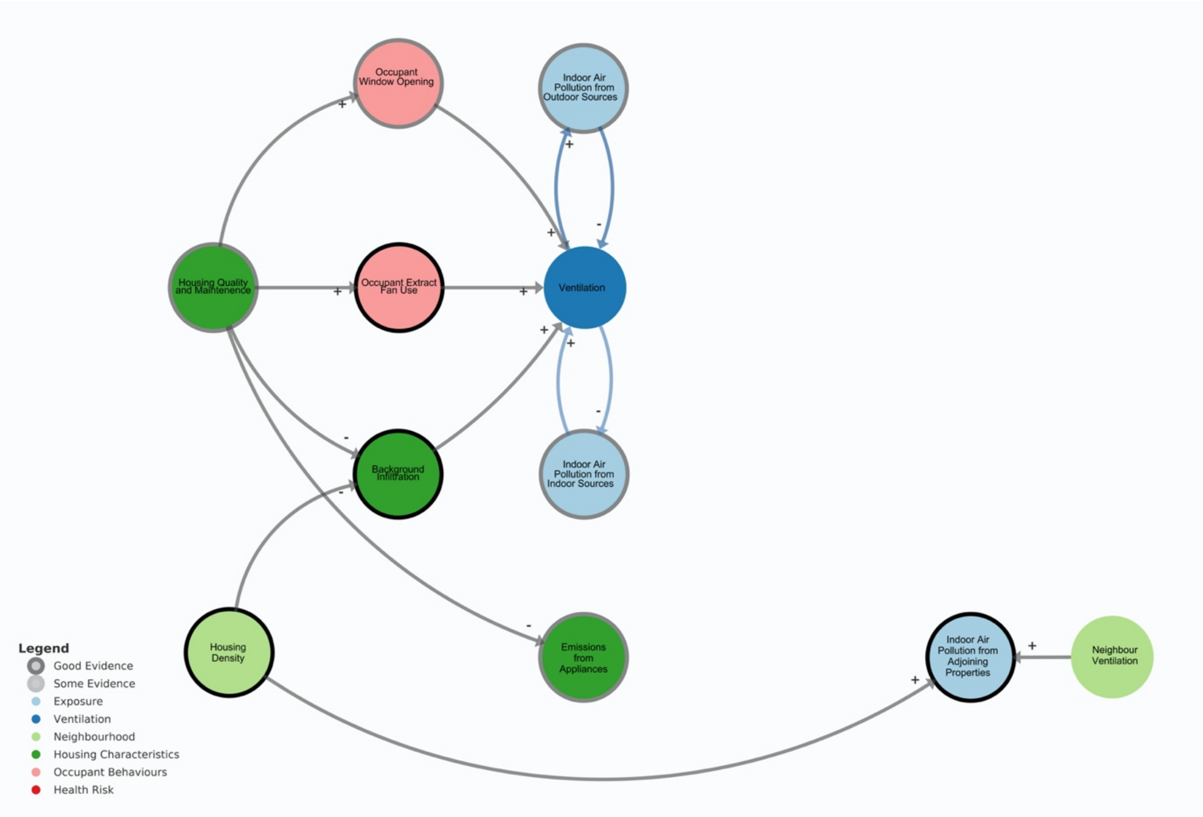


**Figure S2.** The relationship between housing quality and indoor air pollution, described in Section 2.2.

Variables with black outlines are those which have empirical evidence to support socioeconomic differences in levels in London, while those with grey outlines have only limited data to support the relationship. Summarised evidence indicates lower frequencies of working extract fans, reduced infiltration rates, and greater housing density which can limit the number of openable windows and can lead to pollution moving between adjoining dwellings. There is some limited evidence to suggest that some pollution-emitting appliances may be less well maintained.

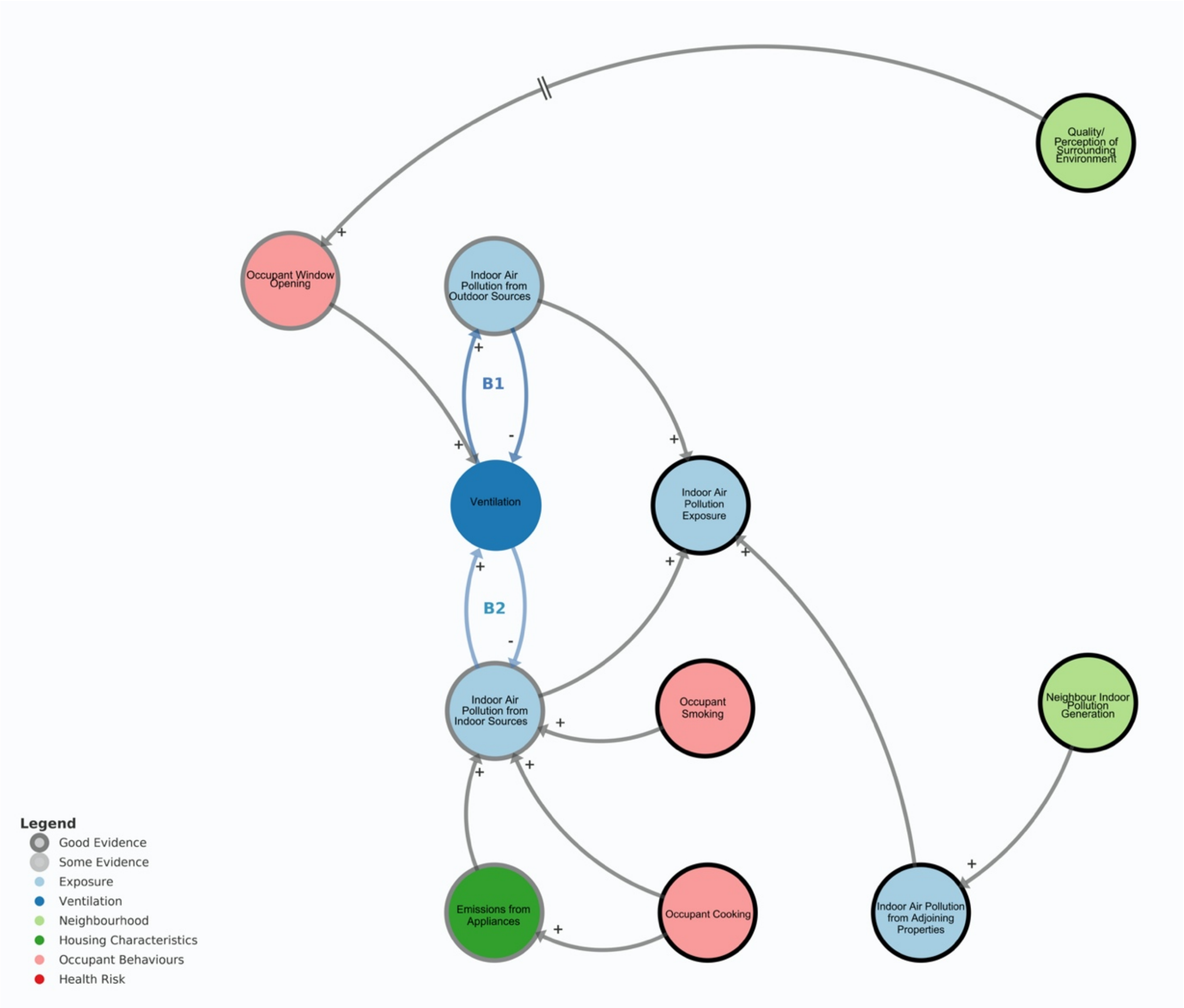


**Figure S3.** The relationship between occupant behaviours and indoor air pollution, described in Section 2.3.

Variables with black outlines are those which have empirical evidence to support socioeconomic differences in levels in London, while those with grey outlines have only limited data to support the relationship. There is evidence that low SES households are more likely to have a smoker resident, and that low SES households may spend longer cooking indoors to accommodate larger households. There is limited evidence that suggests that low SES households may open their windows less due to security concerns.

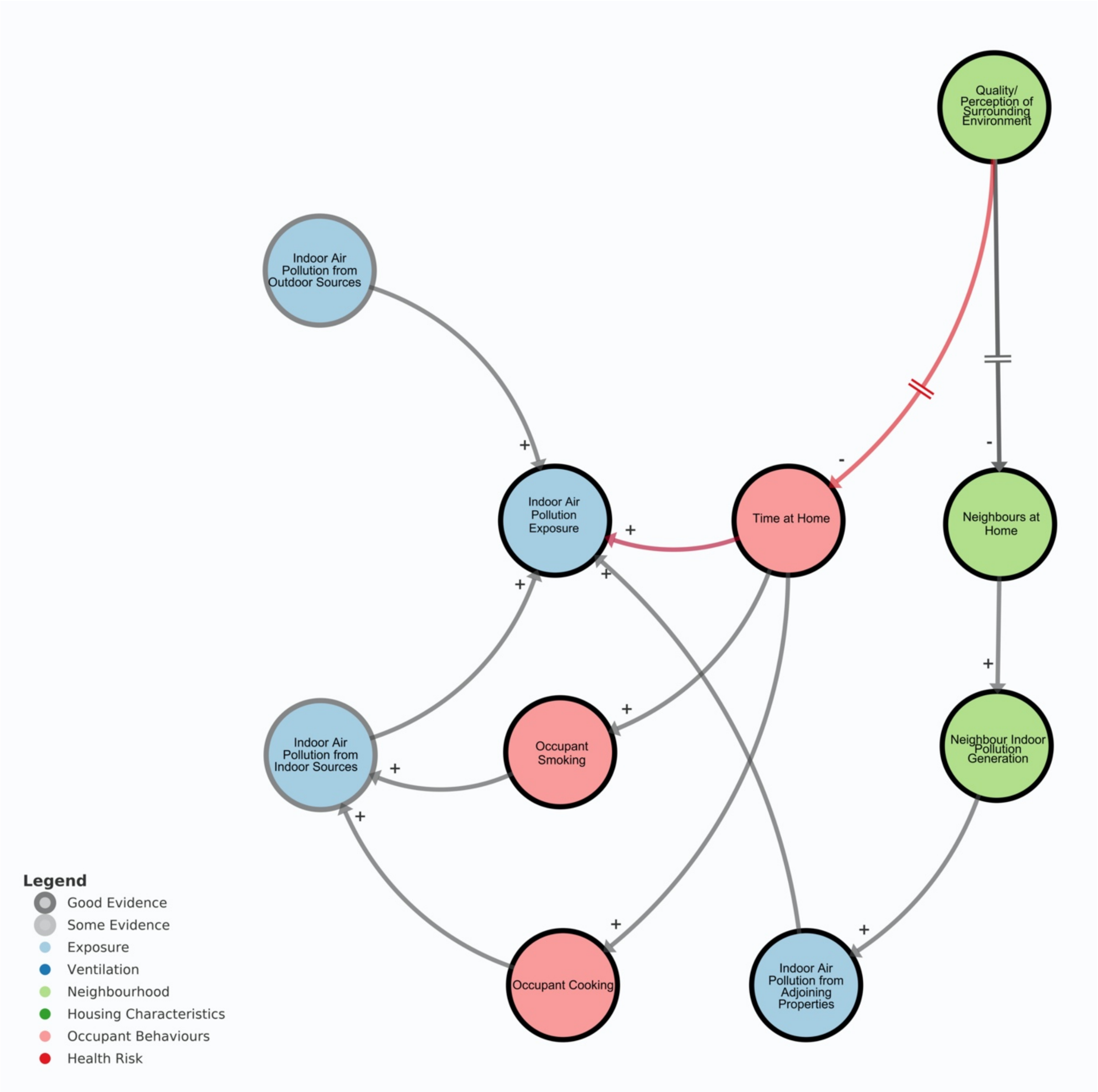


**Figure S4.** The relationship between occupant time at home and indoor air pollution exposure, described in Section 2.4.

Variables with black outlines are those which have empirical evidence to support socioeconomic differences in levels in London, while those with grey outlines have only limited data to support the relationship. The analysis of the time activity data indicates that low SES individuals spend a greater amount of time at home. This may be driven by the lower perception or quality of the surrounding environment.

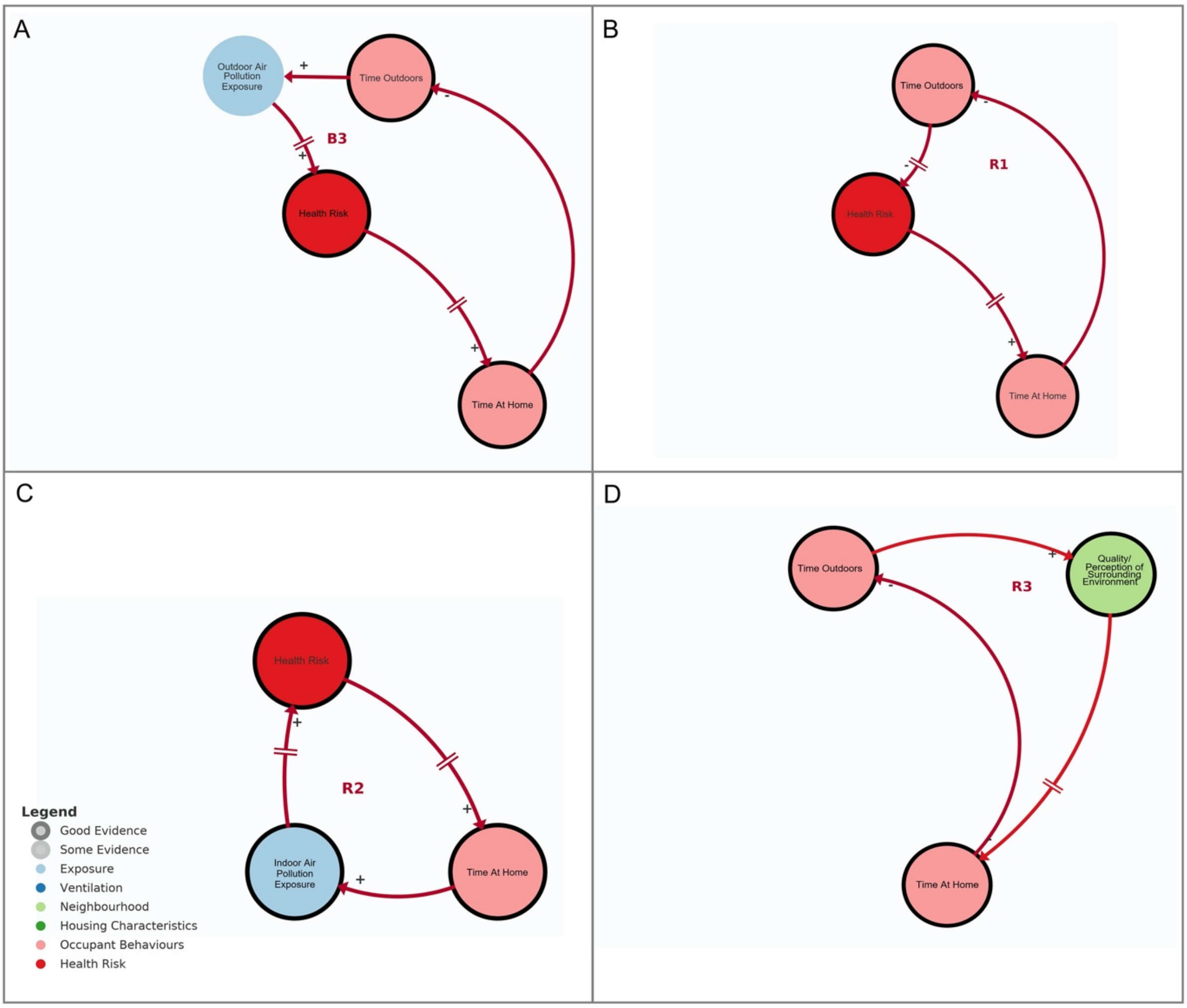


**Figure S5.** The relationship between occupant health air pollution exposure, described in Section 2.5.

Variables with black outlines are those which have empirical evidence to support socioeconomic differences in levels in London, while those with grey outlines have only limited data to support the relationship.

S5(a): Balancing loop B3 shows how increased exposures to outdoor air pollution can gradually lead to increased health issues, resulting in individuals spending more time at home and a consequent reduction in outdoor air pollution exposures. Evidence has been summarised showing SES disparities in time at home and underlying health issues.

S5(b): Reinforcing loop R1 that shows how an increased time at home may decrease the time spent outdoors, gradually increasing health risks due to – for example – a lack of physical activity, which further increases time at home.

S5(c): Reinforcing loop R2 illustrates how increased time at home due to air-pollution related health problems reinforces exposures to indoor air pollution. Therefore, as health issues due to indoor and outdoor air pollution increase, the exposure balance shifts towards the indoors from the outdoors.

S5(d): Reinforcing loop R3 describes how a low perception of their local environment may gradually lead to individuals spending more time indoors, further reducing their perception of their local environment.
